# Simultaneous assessment of the synthesis rate and transcapillary escape rate of albumin in inflammation and surgery

**DOI:** 10.1186/s13054-016-1536-6

**Published:** 2016-11-15

**Authors:** András Komáromi, Ulrika Estenberg, Folke Hammarqvist, Olav Rooyackers, Jan Wernerman, Åke Norberg

**Affiliations:** 1 Department of Clinical Science, Intervention and Technology (CLINTEC), Karolinska Institutet, Stockholm, Sweden; 2 Department of Anesthesiology and Intensive Care Medicine, Karolinska University Hospital, Huddinge, Stockholm, Sweden; 3 Department of Nuclear Medicine, Karolinska University Hospital, Huddinge, Stockholm, Sweden; 4 Center for Digestive Diseases, Karolinska University Hospital, Huddinge, Stockholm, Sweden

**Keywords:** Albumin kinetics, Hypoalbuminemia, Capillary leakage

## Abstract

**Background:**

Better knowledge of albumin kinetics is needed to define the indications for albumin use in clinical practice. This study involved two approaches: the synthesis rate and transcapillary escape rate of albumin were measured simultaneously at different levels of plasma albumin concentration in relation to acute inflammation and surgery; and two different tracers were compared to determine plasma volume and the transcapillary escape rate.

**Methods:**

Healthy volunteers (*n* = 10), patients with acute inflammatory abdominal disease (*n* = 10), and patients undergoing elective pancreatic resection (*n* = 10) were studied. The albumin synthesis rate was measured by the incorporation of deuterium-labeled phenylalanine. Plasma volume and the transcapillary escape rate were assessed using ^123^I-labeled and ^125^I-labeled albumin.

**Results:**

A 50 % elevated de-novo albumin synthesis rate was seen in patients with acute inflammation and marked hypoalbuminemia, while patients with marginal hypoalbuminemia before the start of surgery had a normal albumin synthesis rate. The transcapillary escape rate was elevated intraoperatively during the reconstructive phase of pancreatic surgery, when plasma albumin was decreased but stable. In acute inflammation with marked hypoalbuminemia, the transcapillary escape rate was no different from normal. ^123^I-labeled and ^125^I-labeled albumin were found exchangeable for plasma volume determinations, but could be used only in groups of patients for the transcapillary escape rate.

**Conclusions:**

This observational study illustrates the limited information contained in albumin plasma concentrations to reflect albumin kinetics. On the contrary, single measurements of the synthesis rate and/or transcapillary escape rate of albumin obviously cannot explain the plasma level of albumin or the changes seen in plasma albumin concentration.

**Trial registration:**

www.clinicaltrials.gov, study number NCT01686776. Registered 13 September 2012.

## Background

Exogenous albumin is used extensively in clinical practice, for hemodynamic stabilization and to treat hypoalbuminemia [1]. There are controversies around the use of exogenous albumin and aggregated evidence in meta-analyses shows conflicting results in critically ill patients [2–4]. To enable studies of albumin turnover in states with relatively rapid changes in plasma albumin concentration, it is helpful to employ techniques that reflect both the synthesis and distribution of albumin. To our knowledge, a report of simultaneous assessment of both these aspects of albumin kinetics has been published only recently [5].

Although acute catabolism and inflammation are associated with a low plasma albumin concentration, there is simultaneously a high synthesis rate of albumin [6, 7]. This is in contrast to other states of hypoalbuminemia, like malnutrition and liver failure, when there is a low synthesis rate of albumin [8]. The albumin in-vivo synthesis rate is measured quantitatively by the incorporation of isotope-labeled amino acids into albumin. Today stable isotopes are used for labeling. The constant infusion method uses an infusion of a stable isotope-labeled amino acid during 4–12 h and blood sampling during the infusion period, while the flooding technique uses administration of a bolus of isotope-labeled amino acid followed by blood sampling during 90 min [5, 9, 10].

Albumin turnover is heavily influenced by the distribution, in which the transcapillary escape rate (TER) of albumin plays an important role. The TER is 5 %/hour in healthy subjects in the basal state, but increases in sepsis and following surgery [11]. Iodine-labeled albumin is used for measurement of the TER. Several different radioactive iodine tracers with different radiation and half-lives may be utilized [5]. In a dynamic situation it may be advantageous to make multiple measurements over time. Repeated measurements employing the same tracer necessitate an increase in dose. Use of several tracers opens the possibility to keep the radiation dose low.

This study was designed to compare plasma volume (PV) and the TER obtained with albumin labeled with two different radioactive tracers, ^125^I and ^123^I, to determine whether they are exchangeable for repeated measurements in longitudinal studies. An additional aim was to simultaneously assess the albumin synthesis rate and albumin TER in clinical settings with variable plasma albumin concentration, including nonstable situations. Hence albumin kinetics was assessed in three different subject groups: healthy volunteers (group A), patients with an acute abdominal inflammatory state (group B), and patients during extensive pancreatic surgery (group C).

## Methods

### Materials

L-ring-^2^H_5_ phenylalanine, 99 atom percent (Cambridge Isotope Laboratories Inc., Andover MA, USA), was dissolved in sterile water together with unlabeled phenylalanine to a concentration of 20 mg/ml, 10 molar percent excess. The solutions were prepared, filter-sterilized, and stored in sterile 100-ml containers by APL Pharma Specials (Huddinge, Sweden).

Commercial ^125^I-labeled albumin 185 kBq/ml was purchased (Seralb-125^©^; IBA molecular, Gif-sur-Yvette Cedex, France). ^123^I-labeled albumin was prepared in the Department of Nuclear Medicine at Karolinska University Hospital, Huddinge, Sweden by incubation of sodium iodine (^123^I, 37 MBq/ml; Mallinckrodt Medical) with Albunorm 200 mg/ml (Octapharma, Stockholm, Sweden) and Pierce Iodination Beads (product number 28665; Thermo Fisher Scientific Inc., Waltham, MA, USA), and then filtering through a gel column for separation of free iodine (product number 17-0851-01, PD-10 Desalting column; Amersham Biosciences, Uppsala, Sweden). The final preparation contained less than 2 % free iodine.

### Subjects

In total, 30 subjects in three groups were studied: (group A) 10 healthy volunteers, (group B) 10 hospitalized surgical patients with acute inflammatory abdominal disease and C-reactive protein > 100 mg/ml; and (group C) 10 patients undergoing elective pancreatic resection due to suspected malignancy. Exclusion criteria in all groups were age < 40 years (based on a recommendation by the World Health Organization to abstain from thyroid protection at low radio iodine doses [12]), pregnancy, and hypersensitivity to the study drug (iodinated albumin). Pregnancy tests were performed in women < 55 years old. The nature, purposes, and potential risks of the experimental procedures were explained to the patients before obtaining their voluntary informed consent in writing. The study protocol conformed to the ethical guidelines of the 2008 Declaration of Helsinki and received a-priori approval by the Ethics Review Board in Stockholm and the Swedish Medical Products Agency (EudraCT 2012-002638-35, July 31, 2012). The study was also registered at www.clinicaltrials.gov, study number NCT01686776 (September 13, 2012) and monitored by Karolinska Trial Alliance.

The healthy volunteers in group A were investigated in the postabsorptive state in the supine position. They were allowed to drink tap water.

Group B patients were investigated lying in their beds, receiving i.v. fluid therapy, Ringer’s acetate 1–2 ml/kg/h, pain relief, and antibiotics when indicated. Four patients in this group had acute cholecystitis, three patients had acute pancreatitis, and three patients had perforated appendicitis. Six of these patients had surgery 1–4 days before the study procedure (three cholecystectomies, three appendectomies). The following comorbidities were noted: diabetes mellitus in two patients, gout in one patient, alcohol abuse in one patient, and previous stroke in one patient.

**Fig. 1:**
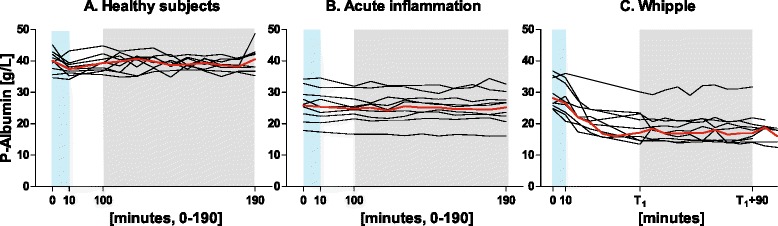
Plasma albumin concentration over time. **a** Healthy volunteers (*n* = 9). **b** Surgical patients with acute inflammation (*n* = 10). **c** Patients undergoing pancreatic resection (*n* = 9). *Green area*, albumin synthesis measurements; *gray area*, measurement of the TER and PV. *x* axes are not linear and are different in the three panels. In **c** *T*_*1*_ denotes the start of the reconstruction phase during surgery, 277 ± 94 min after the beginning of the surgical procedure. Concentrations of all individual patients are given in the three panels and the median values are indicated (*red line*). On a group level, plasma albumin concentration was stable in healthy subjects and patients with acute inflammation, while plasma albumin concentration decreased until the start of the reconstruction phase during pancreatic surgery. *P-albumin* plasma albumin, *Whipple* pancreatic surgery

Six patients in group C suffered from pancreatic cancer, two patients from intraductal papillary mucinous neoplasm, one patient from duodenal cancer, and one patient from a cystic tumor in the pancreas that turned out to be benign. The patients had a number of comorbidities apart from their tumors: hypertension in two patients, diabetes mellitus in one patient, atrial fibrillation in one patient, obesity in one patient, and distal muscular dystrophy in one patient. These patients received general anesthesia (induction with propofol-fentanyl, maintenance with sevoflurane-fentanyl). All patients also received an epidural catheter and arterial and central venous lines. Perioperative pain relief was provided with continuous epidural infusion of an isotonic saline solution containing bupivacaine 1 mg/ml, fentanyl 2 μg/ml, and adrenaline 2 μg/ml (APL Pharma, Stockholm, Sweden). In this patient group the albumin synthesis measurement was performed immediately after induction of general anesthesia, and the TER measurement after removal of tumor and hemostasis, in a stable, nonbleeding reconstructive phase (Fig. 1c). The intraoperative fluid therapy management was 1 ml/kg/h of isotonic buffered glucose 25 mg/ml, Ringer’s acetate 2 ml/kg/h, and starch 2 ml/kg/h (Volulyte®; Fresenius Kabi, Bad Homburg, Germany). Bleeding substitution was given at the discretion of the attending anesthesiologist.

### Study protocol

All subjects were monitored with continuous ECG, and pulse oximetry during albumin synthesis, PV, and TER measurements. Groups A + B were monitored with noninvasive blood pressure recordings, while patients in group C were monitored with invasive arterial blood pressure measurement and capnography.

In group A individuals, antecubital venous lines were inserted bilaterally; one for administration of Ringer’s acetate 1–2 ml/kg/h and study-drug administration, and the other line for blood sampling. Subjects in group B already received an infusion of Ringer’s acetate 2 ml/kg/h by a peripheral venous line also used for study-drug administration, and a second venous line was inserted in the opposite arm for blood sampling. In group C patients, catheters in use for the anesthetic procedure were used, an arterial line for blood sampling, and a peripheral venous line for study-drug administration.

All subjects received an infusion of L-^2^H_5_ phenylalanine (10 molar percent excess, 45 mg/kg) during 10 min and blood samples were taken during 90 min for albumin synthesis assessment, at 0, 5, 10, 15, 30, 50, 70, and 90 min after the start of infusion. Blood samples were centrifuged at 2000 × *g* and plasma was kept at −80 °C until analysis.

After the albumin synthesis assessment was completed, ^123^I-albumin 2 MBq and ^125^I-albumin 200 kBq were injected for determination of the TER and PV including blood sampling every 10 min for 90 min. The total effective radiation dose for the subjects was <0.5 mSv, which is less than 50 % of the natural background radiation during 1 year (average in Sweden: 1.1 mSv/year [13]). According to the World Health Organization, prophylactic administration of stable iodine is not indicated to protect the thyroid gland at such low doses for individuals over 40 years old [12].

### Transcapillary escape rate and plasma volume determinations

Each plasma sample was analyzed in a gamma counter (Wallac Compugamma CS1282) during 15–90 min per sample to achieve a total of at least 10,000 counts to keep the error caused by variability in counts measured below 1 %. All values were then back extrapolated to the time point when the isotope was injected into the subject. In order to calculate the PV and TER we made the following assumptions: steady state of albumin metabolism during the period of investigation (90 min); both tracers, ^123^I-HSA and ^125^I-HSA, behave like endogenous albumin; the extravasation rate of both tracers is monoexponential; and the tracers are stable and their catabolism during the period of investigation is negligible. After logarithmic transformation of plasma counts (counts per minute (cpm)) a linear regression was performed. The plasma activity at *t* = 0 was determined from the regression equation. PV was calculated by division of the given dose of tracer (cpm) with the plasma activity (cpm/ml) at *t* = 0. The TER was assessed from the slope of the regression line.

The three groups have very different composition concerning gender and age. To make a comparison of the PVs between the groups possible, therefore, the measured PVs are expressed in relation (%) to the calculated ideal PV from anthropometric measures [14].

### Albumin fractional and absolute synthesis rate determinations

Measurement of the synthesis rate of albumin employing the flooding technique with L-^2^H_5_ phenylalanine has been described previously in detail [15]. Briefly, albumin in plasma samples was isolated using acid ethanol extraction, followed by extensive washing in order to remove traces of free phenylalanine and then hydrolysis with HCl. After enzymatic conversion into phenylethylamine, the enrichment of L-^2^H_5_ phenylalanine from albumin hydrolysates was determined by monitoring the ions, at m/z 180 and 183, of the t-butyldimethylsilyl derivative of phenylethylamine in comparison with known standards on a quadrupole gas chromatography mass spectrometer (Agilent, Kista, Sweden). Analysis of plasma-free phenylalanine enrichment was performed after acid precipitation and cation-exchange chromatography by monitoring the ions, at m/z 336 and 341, of the t-butyldimethylsilyl derivative. The fractional synthesis rate (FSR) of albumin (i.e., the fraction of intravascular albumin pool that is synthesized every day) was determined using the formula described previously [16]:

$$ \mathrm{F}\mathrm{S}\mathrm{R} = \left(\mathrm{P}2-\mathrm{P}1\right)/\mathrm{A}\mathrm{U}\mathrm{C} \times 100 $$

where FSR is expressed in percent per day and P1 and P2 represent enrichment of phenylalanine in albumin at two time points, t1 and t2, after the curve of enrichment becomes linear, based on three samples (50, 70, and 90 min). AUC is the area under the curve for enrichment of plasma-free phenylalanine between time points t1 and t2 adjusted for the secretion time (i.e., the temporal lag period before the appearance of labeled albumin in plasma). The secretion time was assessed by plotting each individual’s regression line for the linear part of the albumin enrichment curve and extrapolating to the baseline enrichment [16]. The absolute synthesis rate (ASR) for albumin was calculated as the product of the fractional rate of albumin synthesis and the intravascular albumin mass, calculated from the plasma albumin concentration and the measured PV.

### Albumin concentration determinations

Blood samples for determination of plasma albumin were spun at 2000 × *g*. Plasma was transferred to polypropylene vials and frozen at −20 °C until analyzed by nephelometry at Studiecenter Karolinska University Hospital Solna.

### Statistical analysis

Data are presented as mean ± SD or median (range) as appropriate by Shapiro–Wilk’s test of normality. One-way ANOVA is used for comparison of the three groups in terms of PV, ASR, and FSR, followed by the Bonferroni post-hoc test for pair-wise comparisons. Kruskal–Wallis ANOVA for three independent groups is used for the TER, followed by two-tailed multiple comparisons of mean ranks for all groups. Correlations are assessed using Pearson’s coefficient of correlation. The study is powered to be able to compare the TERs measured by albumin labeled with two different isotopes. With *n* = 10, there is an 80 % power to find a difference corresponding to an effect size of one (the difference between paired samples divided by the standard deviation of that difference) using a two-sided test and alpha = 0.05 within each of the studied groups. With a precision in the TER of 2 %/hour as suggested [17], a similar difference of 2 %/hour will be detected by *n* = 10. If all three groups are analyzed together (*n* = 30), a power of 80 % is reached to detect an effect size of 0.53, suggesting that a bias of only 1 % will be found significant. Statistical analysis was performed using STATISTICA 10 (Statsoft Inc, Tulsa, OK, USA).

## Results

The characteristics of the subjects studied—healthy volunteers (group A), surgical patients with acute inflammation (group B), and patients scheduled for pancreatic resection (group C)—are presented in Table 1. Volunteers in group A were younger, had a lower BMI, and had a different gender distribution compared with groups B and C. In group C the median (range) intraoperative blood loss was 450 ml (50–1450 ml), and one patient received 500 ml erythrocyte concentrate transfusion. In this group, nine patients had pancreatoduodenectomy, with one of these also having splenectomy, gastrectomy, hemicolectomy, and aortocaval lymphadenactomy; and one patient had enucleation of a pancreatic cyst. Because of analytic errors, data from one subject in group A and from one subject in group C were excluded from the PV and TER results. Similarly because of missing samples, data from one subject in group C were excluded from the albumin FSR and ASR results. No subjects were excluded for being outliers, as illustrated by the patient in group C with a very high TER who underwent a much more extensive and long-lasting surgical procedure than any other subject.

**Table 1:** Characteristics of subjects

	Healthy volunteers	Acute inflammation	Whipple^a^
Sex, males: females	3:7	8:2	9:1
Age (years)	50 ± 8	62 ± 13	61 ± 11
Body mass index (kg/m^2^)	24.4 ± 2.3	28.9 ± 3.7	26.0 ± 5.6
Plasma C-reactive protein (mg/L)	1.4 (0.9–4)	234 (106–450)	9 (0.9–44)
Plasma albumin (g/L)	39.0 ± 2.0	26.0 ± 4.6	32.8 ± 6.8
White blood cell count (10^9^/L)	6.2 ± 0.9	13.0 ± 5.0	7.0 ± 2.3
Platelet count (10^9^/L)	232 ± 30	232 ± 66	316 ± 120
Prothrombin time, INR^b^		1.0 ± 0.1	1.1 ± 0.2
Plasma creatinine (μmol/L)	71 ± 14	83 ± 26	71 ± 17

Data presented as mean ± standard deviation or median (range) as appropriate according to Shapiro-Wilk’s test of normality

^a^Baseline measurements

^b^International normalized ratio (*INR*), no units, normal range 0.8–1.2

As illustrated in Fig. 1, the plasma albumin concentration was stable for groups A and B, while the plasma albumin concentration decreased between the albumin synthesis measurement and the TER/PV measurements in group C (pancreatic surgery). The time period of albumin concentration decrease started with administration of epidural blockade and general anesthesia, and continued until the end of the dissection part of the surgical procedure.

**Fig. 2:**
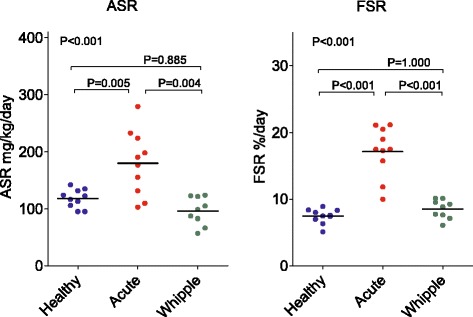
Fractional synthesis rate (*FSR*) and absolute synthesis rate (*ASR*) of albumin in healthy volunteers (*n* = 10, *blue*), in surgical patients with acute inflammation (*n* = 10, *red*), and in patients undergoing pancreatic resection (*n* = 9, *green*) as measured by incorporation of d5-phenylalanine into protein. Median values indicated by *black lines*. Patients with acute inflammation were different from the two other groups for both aspects of the synthesis rate (*P* < 0.001). *Whipple* pancreatic surgery

The albumin synthesis rates for the three different groups are shown in Fig. 2, illustrating the elevated albumin synthesis rate in patients with acute inflammation as compared with the other two groups, both measured in the basal state. Patients with acute inflammation (group B) were different both in FSR and ASR as compared with healthy volunteers and patients scheduled for pancreatic surgery preoperatively (*P* < 0.001).

**Fig. 3:**
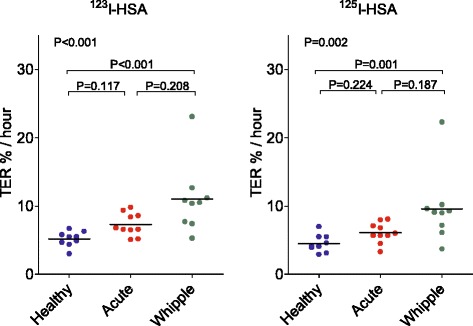
Transcapillary escape rate of albumin (*TER*) in healthy volunteers (*n* = 9, *blue*), in surgical patients with acute inflammation (*n* = 10, *red*), and in patients undergoing pancreatic resection (*n* = 9, *green*) as measured by ^123^I-albumin and ^125^I-albumin, respectively. Median values indicated by *black lines*. The TER is different during pancreatic surgery as compared with controls for both ^123^I-albumin and ^125^I-albumin. *HSA* human serum albumin, *Whipple* pancreatic surgery

**Fig. 4:**
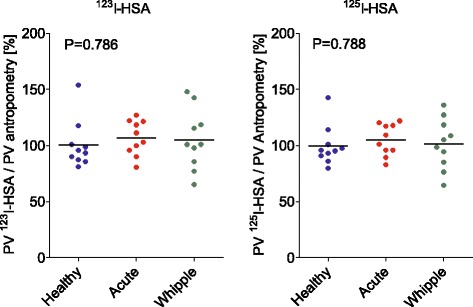
Plasma volume (*PV*) in healthy volunteers (*n* = 9, *blue*), in surgical patients with acute inflammation (*n* = 10, *red*), and in patients undergoing pancreatic resection (*n* = 9, *green*) as measured by ^123^I-albumin and ^125^I-albumin, respectively. To make groups more comparable the measured PVs are related to the anthropometrically calculated PVs [14]. Median values indicated by *black lines*. No difference in PV between the three groups was detected. *HSA* human serum albumin, *Whipple* pancreatic surgery

**Fig. 5:**
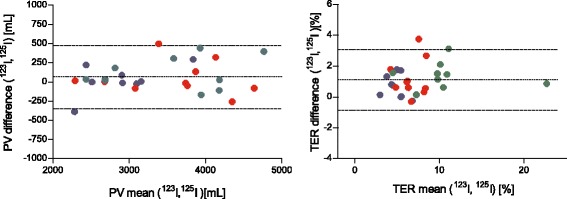
Bland–Altman plots demonstrating agreement between measurements of the transcapillary escape rate (*TER*) and plasma volume (*PV*) as measured by ^123^I-albumin and ^125^I-albumin, respectively, in healthy volunteers (*n* = 9, *blue*), in surgical patients with acute inflammation (*n* = 10, *red*), and in patients undergoing pancreatic resection (*n* = 9, *green*. Limits of agreement given as the mean difference ± 2.045 SD (*n* = 28, *hatched lines*) and the line of identity (*solid line*) are shown

The results using two different albumin labels, ^123^I-albumin and ^125^I-albumin, to determine the TER and PV in the three different groups of subjects are presented in Figs. 3 and 4, respectively, illustrating that the three groups were different in terms of TER but not in terms of PV. Comparing all of the measurements obtained using the two different albumin labels in Bland–Altman plots (Fig. 5) gave a line of identity for PV of 65 ml, but a statistically significant systematic bias of 1.1 %/hour for the TER (*P* < 0.001, *n* = 28). For PV, the confidence interval was ±10 % in the interval 2300–4700 ml. For the TER, the confidence interval was 2 % in the interval 4–11 %/h.

## Discussion

This observational study illustrates the limited information contained in the albumin plasma concentration, when albumin kinetics is considered. The three groups of subjects studied differ in terms of plasma concentration, synthesis rate, and TER. A “snapshot” measurement of the albumin synthesis rate and/or TER obviously cannot explain the plasma level of albumin or the changes seen in plasma albumin concentration.

The albumin synthesis rates for the three groups of subjects compare well with earlier studies. Both volunteers [15] and cholecystitis patients [6] have been investigated previously. The temporal pattern of plasma albumin concentration in patients undergoing pancreatic resection fully reproduced a previous report [18]. The impact on the de-novo synthesis rate during anesthesia and surgery is less well studied, however. Immediately after induction of general anesthesia, a slight decrease in FSR by 1.1 %/24 h is reported in patients undergoing laparoscopic cholecystectomy [19]. The combined effect of general anesthesia and epidural anesthesia has so far not been characterized. In particular, the immediate effect of a major surgical procedure, dramatically increasing the TER, on albumin synthesis is not yet elucidated. It must be noted that the start of the decrease in plasma albumin concentration may initially at least partly be attributable to a transiently expanded PV during the synthesis measurement period, related to the relatively high rate of fluid administration in accordance with the fluid protocol applied. This suggestion is purely hypothetical, however, because we did not measure PV until later, during the reconstructive part of the surgical procedure, when it was no different from the PV in healthy subjects or patients with acute inflammation—although the interindividual scatter was larger, possibly related to the differences in fluid administration (Fig. 4).

The TER was demonstrated to be markedly elevated already during the latter part of a major surgical procedure, at a time point when the full decrease in plasma albumin concentration had occurred. This finding may be interpreted as an indication of rapid changes in the TER during at least the early phase of generalized inflammation in response to major surgery. Despite this, no further decrease in plasma albumin level was seen after this time point (Fig. 1). Further on, 48 hours postoperatively, with a similar decrease in plasma albumin concentration, the TER is back to normal [5, 18]. Nevertheless, our data provide new information regarding the relation between albumin plasma concentration and the TER in surgical trauma. In group B a state of acute inflammation was accompanied by a concomitant low but stable plasma albumin concentration. However, the moderate increase of the TER did not reach statistical significance (Fig. 3). In the early phase of the inflammatory insult, these subjects may have had a transient increase in the TER, no longer present at the time of measurement. The TER reflects the escape of isotopic label from plasma, but the return of albumin to plasma, mainly through the lymphatic system, is not easy to quantify by existing techniques in human subjects. Some information on this possible mismatch between escape and return may be gained by utilizing calculations of fluid and albumin balances [18].

The two different radio-iodine labels of albumin gave identical results for PV, while there was a 1.1 %/hour absolute difference for the TER. This means that the two labels are fully exchangeable for multiple PV determinations, while the exchangeability for TER is limited to situations when absolute differences >4– 5 % are expected. The limits of agreement indicate a standard deviation of <10 % in relative terms, which translates into a possibility to detect differences of this magnitude with a statistical power of 80 % in 11 subjects. The use of three different groups of subjects with, when combined, a larger than normal range of the TER makes this assumption valid in this wider range (4–11 %/hour).

The differences in the TER related to the isotopic label were most likely attributable to the presence of free label in the preparations. Differences attributable to the difference in isotopic half-life were eliminated by the analytic procedure used.

The strengths of this study are the inclusion of three well-defined groups of subjects with different characteristics of plasma albumin concentration, albumin synthesis rate, and TER; and the systematic comparison of two different radio-iodine labels for possible use when multiple determinations of the TER or PV are needed. The limitations of the study are that there is some mismatch in anthropometry between the groups, and that the inclusion criteria in the pancreatic surgery group, which also included surgical outliers, are perhaps too pragmatic.

## Conclusions

This study provides new information on the temporal development of albumin kinetics during major abdominal surgery associated with hypoalbuminemia and an increase in capillary leakage. The results illustrate the limited information contained in albumin plasma concentrations to reflect albumin kinetics. On the contrary, occasional measurements of the synthesis rate and/or TER of albumin obviously cannot explain the plasma level of albumin or the changes seen in plasma albumin concentration. Furthermore the possibilities to use radio-iodine albumin for multiple assessments in studies over albumin kinetics were explored. The labels were found exchangeable for PV determinations, but for the TER only in groups of patients.

## Key messages

- Albumin plasma concentrations poorly reflect albumin kinetics.
- Single measurements of the albumin synthesis rate and/or transcapillary escape rate of albumin poorly explain the plasma level of albumin.
- The transcapillary escape rate is elevated at the end of a major surgical procedure.
- ^123^I-labeled and ^125^I-labeled albumin are exchangeable for plasma volume determinations, but not for transcapillary escape rate measurements.

## Abbreviations

ANOVA: Analysis of variance
ASR: Absolute synthesis rate
AUC: Area under the curve
BMI: Body mass index
FSR: Fractional synthesis rate
PV: Plasma volume
SD: Standard deviation
TER: Transcapillary escape rate
